# Volumetric changes in temporomandibular joint space following trans-oral vertical ramus osteotomy in patients with mandibular prognathism: a one-year follow-up study

**DOI:** 10.1038/s41598-023-51050-y

**Published:** 2024-01-10

**Authors:** Jae-Young Kim, Hae-Seong Yong, Tae-Yeong Kim, Jun-Young Kim, Kug Jin Jeon, Jong-Ki Huh

**Affiliations:** 1 Department of Oral and Maxillofacial Surgery, Gangnam Severance Hospital, Yonsei University College of Dentistry, 211 Eonju-Ro, Gangnam-Gu, Seoul, 06273 Korea; 2 Department of Oral and Maxillofacial Surgery, Yonsei University College of Dentistry, Seoul, Republic of Korea; 3 Department of Oral and Maxillofacial Radiology, Yonsei University College of Dentistry, Seoul, Republic of Korea

**Keywords:** Oral diseases, Dentistry

## Abstract

This study measured and analyzed chronological changes in temporomandibular joint space volume by compartment following transoral vertical ramus osteotomy (TOVRO) using reconstructed 3-dimensional (3D) images of patients with mandibular prognathism. It included 70 joints of 35 patients who underwent TOVRO between January 2018 and December 2021. Computed tomography (CT) or cone-beam CT (CBCT) was performed before surgery (T0) and at 3 days (T1), 6 months (T2), and 12 months postoperatively (T3). These scans were then analyzed using 3D software. The volumes of the overall (Vjs), anterior (Vajs), posterior (Vpjs), medial (Vmjs), and lateral (Vljs) joint spaces were calculated at each time point. A linear mixed model and repeated-measures covariance pattern with unstructured covariance were used to evaluate significant changes in joint space volume over time. Vjs significantly increased to 134.54 ± 34.28 mm^3^ at T3 compared to T0 (*p* < 0.001). Vpjas and Vljs increased by 130.72 ± 10.07 mm^3^ and 109.98 ± 7.52 mm^3^ at T3 compared to T0, respectively (*p* < 0.001). However, no significant difference was observed between T0 and T2 in Vajs and Vmjs (*p* = 0.9999). The observed volume increases in Vpjs and Vljs appeared to contribute to the overall Vjs increase.

## Introduction

Vertical ramus osteotomy (VRO) and sagittal split osteotomy (SSO) stand out as two frequently employed surgical techniques in orthognathic surgery. Specifically, these methods are widely used to correct mandibular prognathism^1^. Notably, VRO, pioneered by Winstanley in 1968^2^, has evolved with technological advancements into a contemporary transoral vertical ramus osteotomy (TOVRO) procedure^3–6^.

During surgery, the periosteum in the lateral and posteroinferior parts of the mandibular ramus is elevated. Immediately after surgery, the mesial bone fragment, which is detached from the surrounding tissues and lacks muscular support, exhibits anterior and downward movements (sagging). The displaced fragment, including the mandibular cotndyle, gradually recovers its original position during the recovery process, assisted by postoperative functional physical therapy^7^.

In a recent study using computed tomography (CT) data, the position and angle of the mandibular condyle displaced immediately after surgery gradually recovered until 6 months post-surgery but did not completely revert to their pre-surgical state^8,9^. Condylar sagging causes temporomandibular joint (TMJ) space alterations, significantly impacting postoperative stability and TMJ disease symptoms following TOVRO^10,11^. However, there is insufficient information regarding these changes occurring within the joint space.

Kim et al.^12^ measured the volume of the TMJ space using reconstructed CT images, and Zhang et al.^13^ demonstrated the reliability of measuring the TMJ space using cone beam CT (CBCT). Three-dimensional (3D) images are free of distortions, such as enlargement or reduction, sometimes observed in 2-dimensional images, and exhibit no overlap between anatomical structures. Consequently, a more detailed analysis of the temporomandibular joint space is possible^14^.

The purpose of this study was to measure and analyze the tendency of volumetric changes in the TMJ space by compartment after TOVRO using reconstructed 3D images of patients with mandibular prognathism.

## Results

Among the 35 patients (mean age: 23.29 ± 5.28), 19 were male and 16 were female. Twenty-nine patients (82.9%) underwent TOVRO with a Le Fort I osteotomy, and six (17.1%) underwent TOVRO alone. A total of seventy TMJ space volumes were analyzed. The results obtained on postoperative day 3 (T1) were estimated average values using mixed model analysis based on data from only 19 patients.

### *Changes in overall Vjs over time (*Fig. 1a*, *Table 1*)*

**Figure 1:**
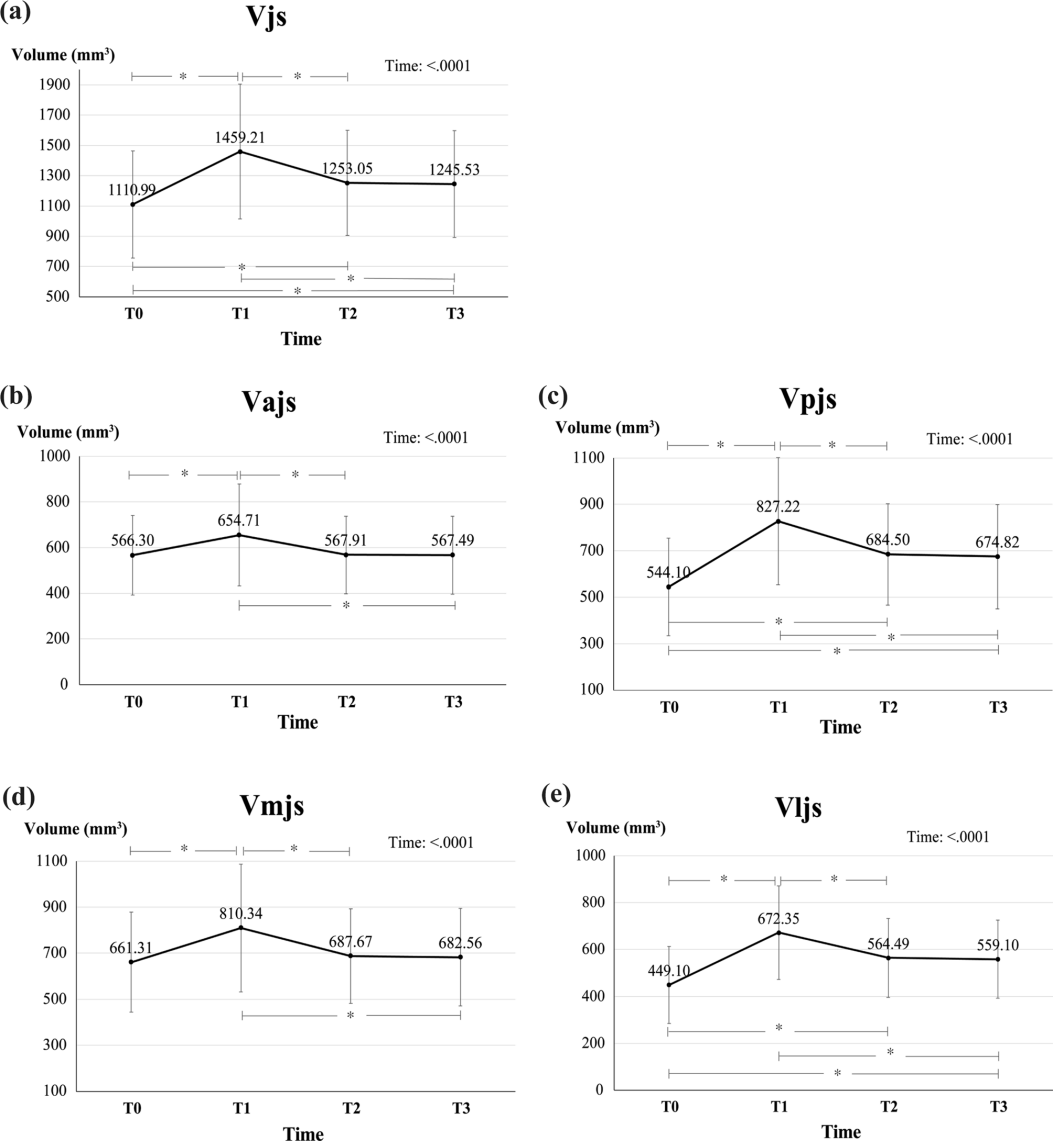
Volumetric changes of joint spaces over time.(**a**) Vjs, the volume of the joint space; (**b**) Vajs, the anterior joint space; (**c**) Vpjs, the posterior joint space; (**d**) Vmjs, the mesial joint space; (**e**) Vljs, the lateral joint space.

**Table 1:** Changes of overall temporomandibular joint space volume (Vjs) over time.

Outcome	Time	Estimated mean (SE)	Overall *p*-value	Post-hoc	Differences (SE)	*p*-value (bonferroni)
Vjs (mm^3^)	T0	1110.99 (49.48)	< .0001	T0 versus T1	348.29 (41.27)	< .0001*
	T1	1459.21 (64.45)		T0 versus T2	142.06 (32.20)	< .0001*
	T2	1253.05 (48.44)		T0 versus T3	134.54 (34.28)	< .0001*
	T3	1245.53 (48.97)		T1 versus T2	− 206.17 (41.58)	< .0001*
				T1 versus T3	− 213.69 (39.12)	< .0001*
				T2 versus T3	− 7.52 (28.48)	> .9999

Vjs, the volume of the joint space.

There was a significant increase (348.29 mm^3^, 31.34%) in the volume of the overall joint space (Vjs) between measurements taken preoperatively (T0) and on postoperative day 3 (T1) (*p* < 0.0001). Subsequently, there was a significant decrease between T1 and measurements taken 6 months postoperatively (T2). However, no significant differences were observed between T2 and measurements taken 12 months postoperatively (T3). The results confirmed that the total joint space volume increased by approximately 134.54 mm^3^ (12.11%) at T3 compared to T0 (*p* < 0.0001).

### *Changes in Vajs and Vpjs over time (*Fig. 1b,c*, *Table 2*)*

**Table 2:** Changes of anterior and posterior temporomandibular joint space volume (Vajs and Vpjs) over time.

Outcome	Time	Estimated mean (SE)	Overall *p*-value	Post-hoc	Differences (SE)	*p*-value (bonferroni)
Vajs (mm^3^)	T0	566.30(20.79)	< .0001	T0 versus T1	88.41 (10.43)	< .0001*
	T1	654.71(24.16)		T0 versus T2	1.61 (7.41)	> .9999
	T2	567.91(20.33)		T0 versus T3	1.19 (7.03)	> .9999
	T3	567.49(20.36)		T1 versus T2	− 86.80 (11.78)	< .0001*
				T1 versus T3	− 87.21 (11.32)	< .0001*
				T2 versus T3	− 0.42 (5.64)	> .9999
Vpjs (mm^3^)	T0	544.10(24.99)	< .0001	T0 versus T1	283.12 (12.83)	< .0001*
	T1	827.22(32.27)		T0 versus T2	140.40 (9.23)	< .0001*
	T2	684.50(26.08)		T0 versus T3	130.72 (10.07)	< .0001*
	T3	674.82(26.75)		T1 versus T2	− 142.72 (12.88)	< .0001*
				T1 versus T3	− 152.40(12.11)	< .0001*
				T2 versus T3	− 9.69 (6.23)	0.7468

Vajs, the anterior joint space; Vpjs, the posterior joint space.

The anterior joint space (Vajs) showed a significant increase of approximately 88.41 mm^3^ (15.61%) between T0 and T1 but decreased thereafter. There were no significant differences between T0 and T2 or between T0 and T3.

The posterior joint space (Vpjs) showed a significant increase (283.12 mm^3^, 52.03%) between T0 and T1 (*p* < 0.0001). A significant decrease was observed between T1 and T2, whereas no significant change was observed between T2 and T3. Therefore, Vpjs was 130.72 mm^3^ (24.02%) higher at T3 than at T0 (*p* < 0.0001).

### *Changes in Vmjs and Vljs over time (*Fig. 1d,e*, *Table 3*)*

**Table 3:** Changes of medial and lateral temporomandibular joint space volume (Vmjs and Vljs) over time.

Outcome	Time	Estimated mean (SE)	Overall *p*-value	Post-hoc	Differences (SE)	*p*-value (bonferroni)
Vmjs (mm^3^)	T0	661.31 (36.80)	0.0035	T0 versus T1	149.02 (39.95)	0.0023
	T1	810.34 (49.44)		T0 versus T2	26.35 (33.83)	> .9999
	T2	687.67 (35.74)		T0 versus T3	21.25 (33.94)	> .9999
	T3	682.56 (36.24)		T1 versus T2	− 122.67 (42.27)	0.0298
				T1 versus T3	− 127.78 (40.58)	0.0145
				T2 versus T3	− 5.11 (32.71)	> .9999
Vljs (mm^3^)	T0	449.10 (19.57)	< .0001	T0 versus T1	223.26 (9.91)	< .0001*
	T1	672.35 (25.91)		T0 versus T2	115.59 (7.64)	< .0001*
	T2	564.69 (20.15)		T0 versus T3	109.98 (7.52)	< .0001*
	T3	559.10 (19.93)		T1 versus T2	− 107.67 (11.07)	< .0001*
				T1 versus T3	− 113.26 (10.67)	< .0001*
				T2 versus T3	− 5.59 (4.43)	> .9999

Vmjs, the mesial joint space; Vljs, the lateral joint space.

The medial joint space (Vmjs) showed a significant increase of 149.03 mm^3^ (22.53%) between T0 and T1, but decreased thereafter, with no significant difference between T0 and T2 or between T0 and T3.

The lateral joint space (Vljs) demonstrated a significant increase (223.25 mm^3^, 49.71%) between T0 and T1 (*p* < 0.0001). There was a significant decrease between T1 and T2, while no significant difference was observed between T2 and T3. Consequently, Vljs was significantly higher (110.00 mm^3^, 24.49%) at T3 than at T0 (*p* < 0.0001).

## Discussion

Immediately after TOVRO, sagging of the mandibular condyle occurs because the proximal segment of the joint, including the mandibular condyle, is free from the surrounding tissues except for the lateral pterygoid muscle, joint capsule, and ligaments located in the condylar region. The influence of the lateral pterygoid muscle located in the mandibular condyle induces rotational movement of the proximal segment, directing it in an anterior-inward or anterior-inward-inferior direction, with the contact area of the proximal and distal bone fragments serving as the center of rotation^9,15,16^.

Recovery of proximal segment displacement involves osseous healing, cortical bone remodeling between the proximal and distal segments, and rehabilitation of the surrounding tissues through functional physical therapy^7^. However, a recent study indicates that the downward and rotational movements of the mandibular condyle, occurring immediately after surgery, only partially recover to the preoperative state during the recovery process, and complete recovery is not achieved even after one year^8,9^. This study delves deeper into the joint space, categorizing it into anterior (Vajs), posterior (Vpjs), medial (Vmjs), and lateral (Vljs) compartments, aiming to pinpoint the specific locations where these changes manifest.

Based on our results, the volumes of Vpjs and Vljs remained elevated at T2 compared to T0, while there was no difference between Vajs and Vmjs between T0 and T2. These findings are consistent with the results of Huang et al.’s study, where 6 months post-surgery, the anterior joint space exhibited no significant change while the posterior joint space increased^17^. Nagata et al.^18^ also reported on the movement of the condyle according to overall postoperative time changes after IVRO. Similar to this study, it exhibited an immediate increase after surgery that gradually recovered over time. However, unlike this study, Nagata et al. used two-dimensional measurements without segmentation. In addition, Hong et al.^19^ reported that the volume of joint space did not recover even six months after surgery. On the other hand, there were studies that reported no change in joint space or a decrease in posterior joint space compared to pre-surgery levels.^20,21^ These studies targeted patients undergoing SSO, introducing potential variations from our study.

The observed increase in the posterior and lateral joint spaces at T3 is thought to be due to the incomplete recovery of the condyle to its original position. This deviation occurred immediately after surgery in the anterior-medial-inferior direction. The force exerted anteriorly and superiorly, combined with the recovery of the masseter muscle and medial pterygoid function post-surgery^22^, likely influences the direction of recovery.

There was no significant change in the volume of any joint space between 6 and 12 months postoperatively (Tables 1, 2, 3). Predicting the exact timing of the changes in the joint space was challenging because CT images were obtained at 3 days and at 6 and 12 months postoperatively. The authors believe that bone fragment movement after surgery typically occurs within the first 6 months, aligning with previous reports^23,24^. Additionally, tooth movement, which can affect the change in mandibular position during postoperative orthodontic procedures, usually occurs within the initial 6 months due to the regional acceleration phenomenon^25^. Consequently, it may be predicted that alterations in the TMJ space postoperatively will stabilize around the 6-month mark.

Zhao et al. reported that vertical ramus osteotomy has favorable effects on the TMJ in animal studies^26^. Several other studies have also highlighted improvements in TMJ disorders (TMD) following a vertical ramus osteotomy^27,28^. In patients with TMJ disease with concurrent anterior disc displacement, the retrodiscal tissue resides between the glenoid fossa and the mandibular condyle. The displaced retrodiscal tissue, exposed to excessive loads, can trigger an inflammatory response. This inflammatory response in the retrodiscal tissue, rich in nociceptive fibers, significantly influences TMJ pain associated with disc displacement^29^. In this study, both Vjs and Vpjs increased significantly after vertical osteotomy in patients with prognathism. The increased joint space remained stable even at 6 months postoperatively. Such changes in the joint space can alleviate the deleterious load applied to the retrodiscal tissue, suggesting a possible reduction in TMJ pain. However, since the patients participating in this study did not complain of TMD symptoms, and TMD symptoms themselves were not investigated, a cautious approach is required.

There are certain limitations to this study. First, the study did not account for mandibular asymmetry. In 2020, Kim et al. investigated the difference in condylar space change after TOVRO in patients with facial asymmetry and found no significant difference between the deviated and non-deviated sides^8^. However, since patients with mandibular asymmetry may exhibit different effects, a more comprehensive study, considering the presence of mandibular asymmetry, is required. Second, relying on estimated CBCT values on postoperative day 3 could be considered another limitation. However, images were only taken for patients requiring post-operative status assessment and not for all the patients. Third, there were mixed patients who underwent TOVRO alone (17.1%). This study was conducted to investigate changes in temporomandibular joint space after IVRO. Recent studies have combined patients who underwent LeFort I osteotomies with those who did not^30–32^. However, since this may affect the results, future research is needed to distinguish between these patient groups. Moreover, employing computer-aided measurement techniques^33^, or artificial intelligence technology^34^, which has been widely studied recently, can help improve human subjectivity in the future. In this study, CT and CBCT were used for pre- and post-operative analyses. Pellerin et al.^35^ reported that the volume of liver lesions measured by CBCT and CT in rabbit experiments was almost the same size [3.8 ± 1.6 cm^3^ (range, 1.3–7.3 cm^3^) on CBCT and 3.9 ± 1.6 cm^3^ (range, 1.8–7.5 cm^3^) on MDCT]. Trindade et al.^36^ also reported that there was no difference in the volume of periapical lesions measured by CBCT and CT. Additionally, the Mimics program used in this study is reported to be quite accurate compared to other software^37^. Nevertheless, a strength of this study lies in the detailed analysis of volumetric changes in the TMJ space, divided into compartments, in patients who underwent TOVRO.

The volume of the TMJ space at 1-year follow-up was higher than that before surgery, which is thought to be due to the increase in the posterior and lateral joint spaces. In addition, the joint space appeared to have stabilized six months after surgery.

## Materials and methods

This retrospective study involved the medical records of patients who were diagnosed with mandibular prognathism and underwent orthognathic surgery between January 2018 and December 2021 at the Department of Oral and Maxillofacial Surgery, Gangnam Severance Hospital, Seoul, Republic of Korea. The exclusion criteria for this study included the following: (1) patients without CBCT images after surgery; (2) poor or inappropriate image quality; (3) patients with pathological conditions such as degenerative joint disease, condylar osteochondroma, or synovial chondromatosis; and (4) patients with congenital facial anomalies such as cleft lip and palate and hemifacial microsomia.

A total of 35 patients were included in the study. The sample size was calculated using the G-power program based on a previous study^8^ (Power = 90%, α = 0.05). Patients underwent preoperative (T0) CT scans for analysis, diagnosis, and simulation surgery, with the fabrication of a rapid prototype model two months before the operation. All patients underwent CBCT at 6 (T2) and 12 months (T3) post-surgery for postoperative follow-up. Of them, 19 patients underwent additional follow-up CBCT on day 3 (T1) post-surgery to assess the surgical results.

Postoperative management adhered to a predefined protocol^7^. During the two weeks following surgery, intermaxillary fixation was performed using surgical archwires and six elastics. Subsequently, physical therapy, including mouth opening and lateral movement, was carried out using two elastics until maximum mouth opening was restored. The splint was then removed, and postoperative orthodontic treatment was initiated.

This study was approved by the Institutional Review Board of Gangnam Severance Hospital (approval No. 3–2022-0466), and a waiver of written informed consent was granted for this retrospective study. This study was conducted in accordance with the principles of the Declaration of Helsinki for human research.

### *Diagnosis of mandibular prognathism*^38^

Mandibular prognathism was diagnosed using lateral cephalometric radiography. The Frankfort horizontal line (the line connecting the porion and orbital; the FH line) was set as the horizontal reference line. The vertical reference line was set as the line perpendicular to the horizontal FH line passing through the nasion point (N-perpendicular line). Mandibular prognathism was diagnosed when the pogonion was more than 1 mm anterior to the vertical reference line.

### Reconstruction and CT image analysis

CT images were obtained using a Siemens Definition AS + (Siemens, Erlangen, Germany) with constant settings (1 mm slice thickness, 7 s scan time, 120 kV, and 90 mAs). CBCT images were obtained using a PaX-i3D (Vatech Co., Gyeonggi-do, Republic of Korea) with constant settings (21 cm x 19 cm field of view, 0.3 mm slice thickness, 24 s scan time, 106 kV, and 65 mAs). The images were reconstructed and analyzed using Mimics 3D analysis software (Materialise, Leuven, Belgium). This process was done by one oral and maxillofacial specialist (H.S. Yong, 6-year experience).

## Reference points and planes

The nasion (N), orbitale (Or), porion (Po), basion (Ba), crista galli (Cg), lateral lip of eminence (Em), and glenoid fossa (Gf) were used as the reference points.

**Figure 2:**
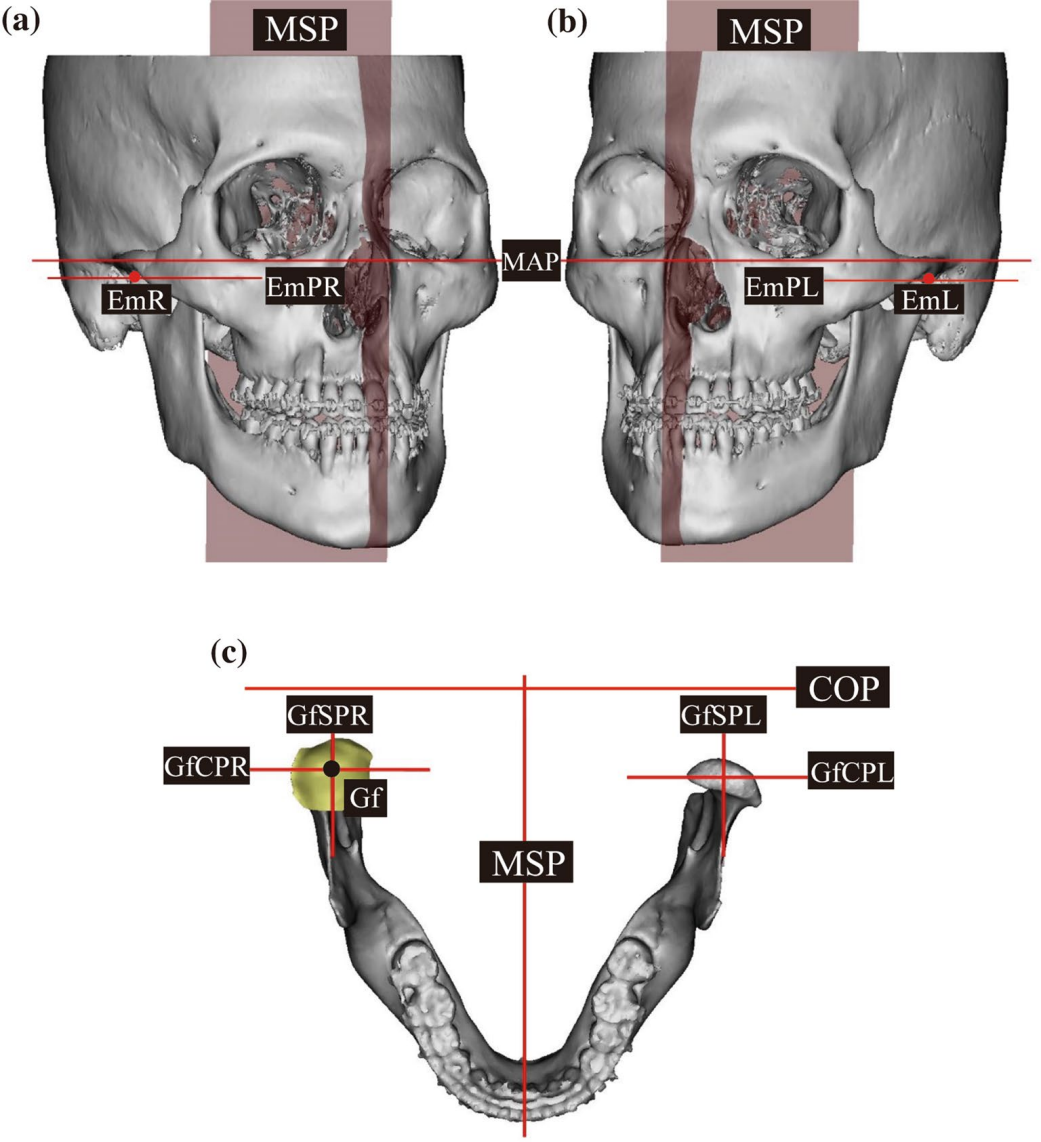
Reference planes for 3D analysis. (**a**) Right side of the skull; (**b**) Left side of the skull EmPR and EmPL are planes parallel to MAP, passing through EmR and EmL. (**c**) occlusal view of the mandible GfSP and GfCP are planes passing through Gf and parallel to MSP and COP, respectively (GfSPR and GfCPR indicate right side, and GfSPL and GfCPL indicate left side). Reconstructed joint space is presented in yellow. MAP, Mid-axial plane; MSP, Mid-sagittal plane; COP, Coronal plane; EmPR, Eminence plane right; EmPL, Eminence plane left; Gf, Glenoid fossa (the most superior point of Glenoid fossa); GfSPR, Glenoid fossa sagittal plane right; GfCPR, Glenoid fossa coronal plane right; GfSPL, Glenoid fossa sagittal plane left; GfCPL, Glenoid fossa coronal plane left.

Based on previous studies, the mid-axial plane (MAP), mid-sagittal plane (MSP), and coronal plane (COP) were established^7^. The MAP was defined as the plane connecting the OrR, OrL, and PoR. The MSP was defined as a plane perpendicular to the MAP passing through the Cg and Ba. The COP was defined as the plane passing through Ba and perpendicular to the MAP and MSP. The plane parallel to the MAP passing through the lateral lip of eminence point (Em) was defined as the eminence plane (EmP). The planes parallel to the MSP and COP and passing through the Gf were defined as GfSP and GfCP, respectively (Fig. 2). The results are summarized in Table 4.

**Table 4:** Reference points and planes for 3D analysis.

Nomenclature (abbreviation)	Definition
Reference points	
Nasion (N)	Intersection point of frontal bone and nasal bone
Orbitale (Or)	The most inferior point of the bony orbitale (Right: OrR, Left: OrL)
Porion (Po)	The most superior point of the external auditory meatus (Right: PoR, Left: PoL)
Basion (Ba)	Midpoint of the anterior margin of the foramen magnum on the occipital bone
Crista galli (Cg)	The most superior point of the crista galli located in the ethmoid bone
Laeteral lip of eminence (Em)	The most inferior and lateral point of articular eminence (Right: EmR, Left: EmL)
Glenoid fossa (Gf)	The most superior point of the glenoid fossa (Right: GfR, Left: GfL)
Reference plane	
Mid-axial plane (MAP)	A plane passing through OrR, OrL, and PoR
Mid-sagittal plane (MSP)	A plane perpendicular to MAP with passing through Cg and Ba
Coronal plane (COP)	A plane perpendicular to MAP and MSP with passing through Ba
Eminence plane (EmP)	A plane parallel to MAP with passing through Em (Right: EmPR, Left: EmPL)
Glenoid fossa sagittal plane (GfSP)	A plane parallel to MSP with passing through Gf (Right: GfSPR, Left: GfSPL)
Glenoid fossa coronal plane (GfCP)	A plane parallel to COP with passing through Gf (Right: GfCPR, Left: GfCPL)

### Superimposition of reconstructed 3D images

The preoperative CT images and 3-day, 6-month, and 12-month postoperative CBCT images were superimposed onto the cranial region.

**Figure 3:**
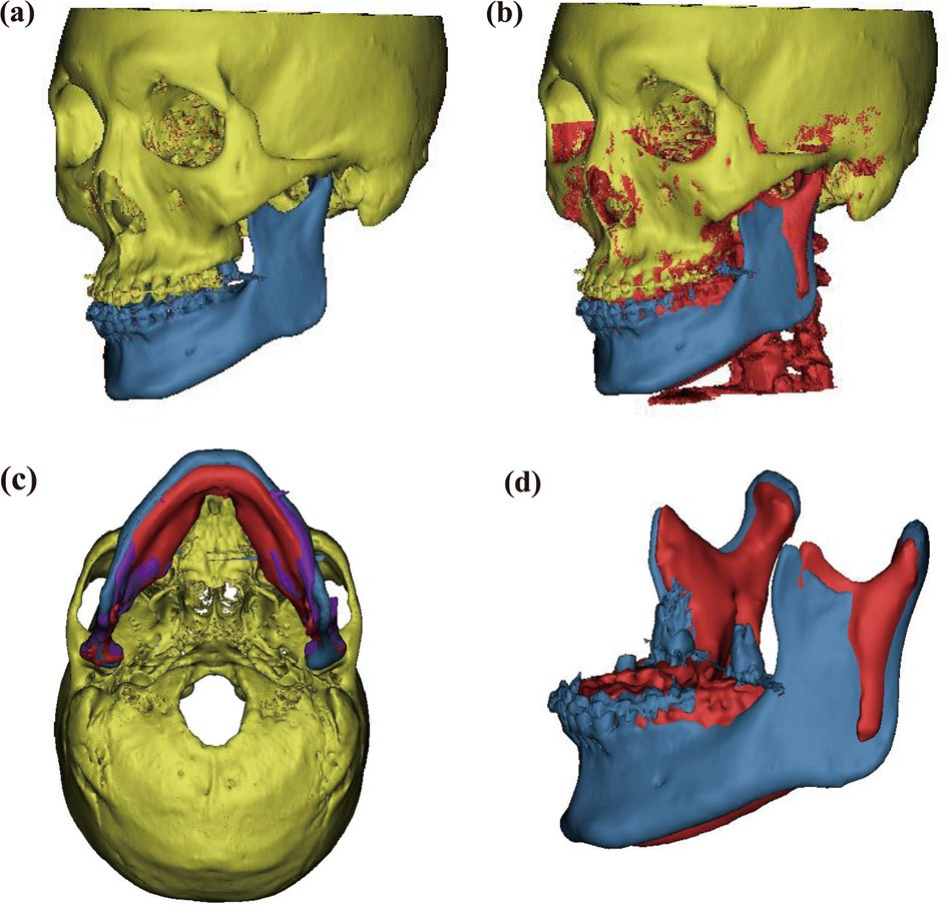
Example of Superimposition of 3D Image. (**a**) Pre-operative image; (**b**) Superimposition of skull between pre-operative and post-operative 6 M images. (**c**) Superimposition of skull among pre-operative, post-operative 3 days, and post-operative 6 M images. (**d**) Superimposition of mandible between pre-operative and post-operative 6 M images (Yellow, skull before surgery; blue, mandible before surgery; purple, post-operative 3 days; red, post-operative 6 months).

The first overlap was performed using the 3-point superimposition method with three reference points (Na, PoR, and Ba) of the skull region, where there was no change in position or shape before and after surgery in the sagittal, coronal, and horizontal cross-sections Additionally, minor discrepancies were rectified so as to make the cranial region superimposition more accurate. An illustrative example of this superimposition is provided in Fig. 3.

### Measurement of volume of joint space on T0

**Figure 4:**
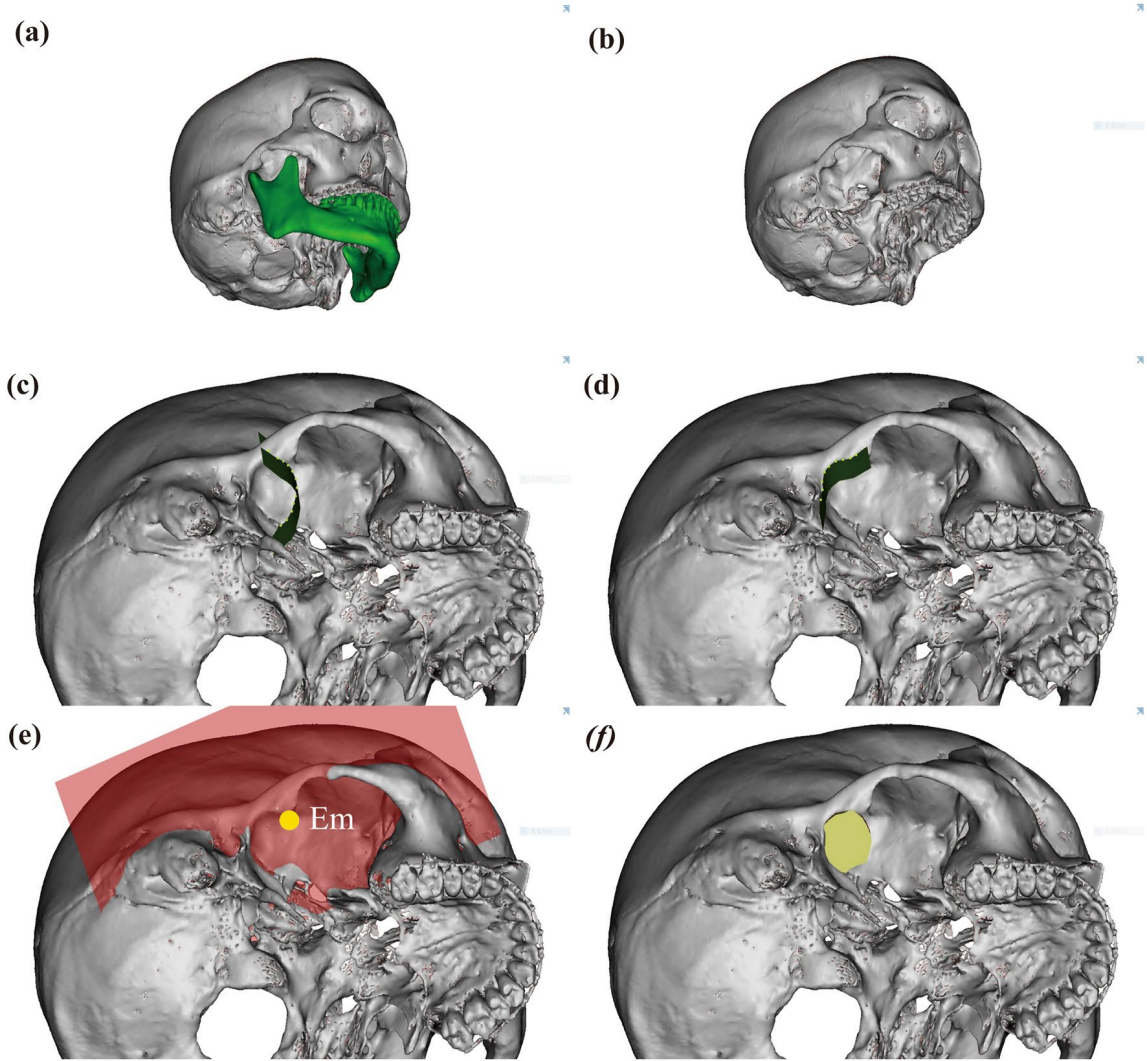
Measurement of volume of the glenoid fossa (Vgf). (**a**, **b**) Separation of the mandible in 3D reconstructed skull; (**c**) The anterior boundary was demarcated by drawing the lowest points of the articular eminence; (**d**) The lateral boundary was demarcated by drawing lateral rim of the articular eminence; (**e**, **f**) The volume of the glenoid fossa (Vgf). The volume above the plane passing through Em and parallel to the MAP was defined as Vgf. Em, the lowest point of the articular eminence; MAP, mid-axial plane.

**Figure 5:**
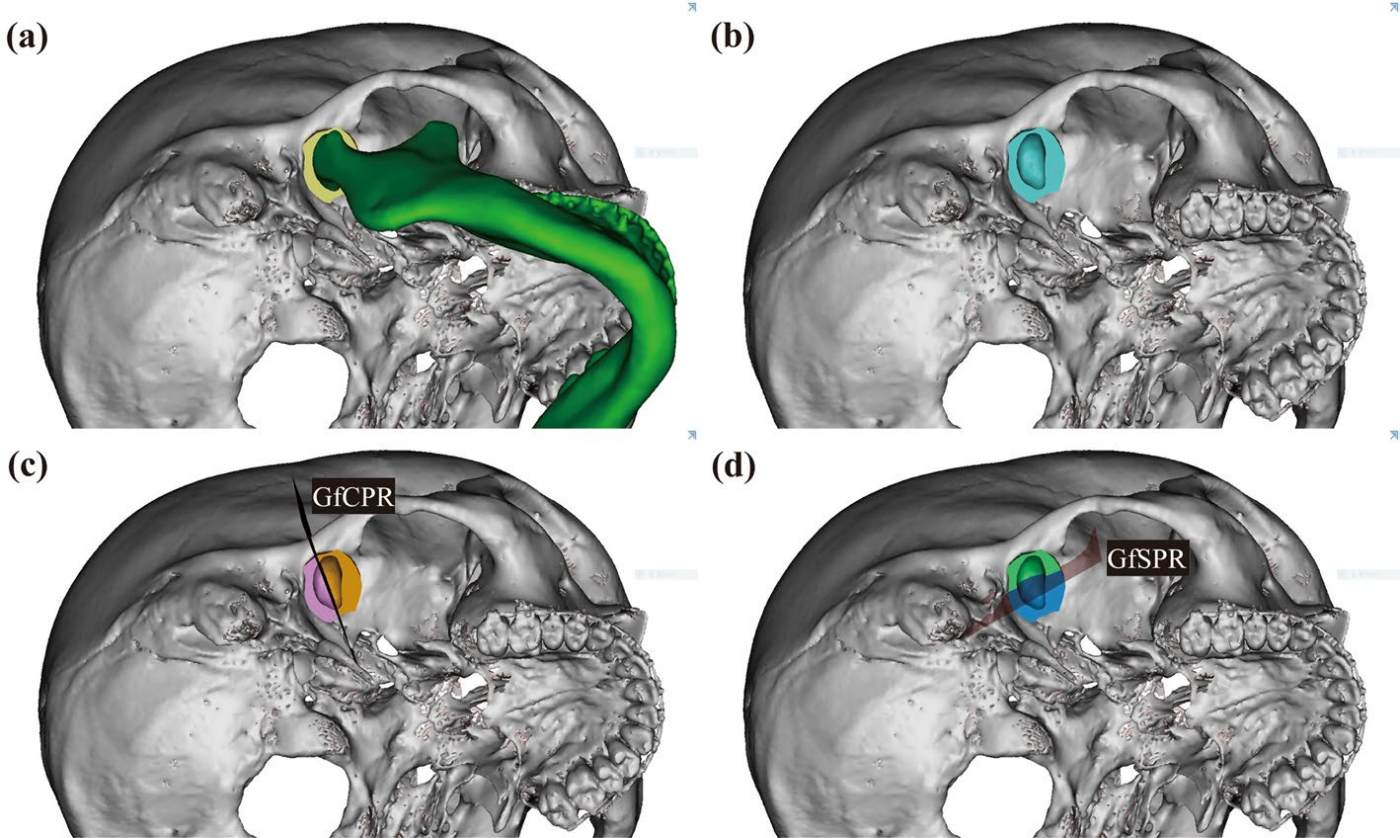
Measurement of volume of the joint space (Vjs). (**a**, **b**) The volume of the joint space (Vjs). The Vjs was measured after removing of reconstructed mandible from Vgf; (**c**) the anterior and posterior joint space (Vajs and Vpjs). Vjs was divided by GfCP for Vajs and Vpjs; (d) The mesial and lateral joint space (Vmjs and Vljs). Vjs was divided by GfSP for Vmjs and Vljs, Vgf, Volume of glenoid fossa; GfSPR, Glenoid fossa sagittal plane right; GfCPR, Glenoid fossa coronal plane right.

In a previous study^11^, the volume of the glenoid fossa (Vgf) was measured first, and the volume of the joint space (Vjs) was measured by removing the condyle. For this study, all the images were captured by H.S. Yong. Vgf and Vjs were calculated as follows:

1. First, the mandible was separated from the 3D-reconstructed skull (Fig. 4a,b).
2. A curved plane connecting the lowest rim of the articular eminence and zygomatic arch was formed at the anterior and posterior boundaries, respectively (Fig. 4c,d).
3. The temporal bone, petrotympanic fissure, and sphenoid bone borders were set as the superior, posterior, and medial borders, respectively.
4. The space bounded above was separated by the eminence plane (EmP), and the volume of the superior space was measured as the volume of the glenoid fossa (Vgf) (Fig. 4e,f).
5. After removing the mandibular condyle from the glenoid fossa formed above, the remaining space was measured as the TMJ space volume (Vjs) (Fig. 5a,b).
6. The TMJ space (Vjs) was subdivided into the glenoid fossa coronal plane (GfCP), and the anterior and posterior parts were measured as the volume of the anterior joint space (Vajs) and posterior joint space (Vpjs) (Fig. 5c).
7. Similarly, the TMJ space (Vjs) was subdivided into the glenoid fossa sagittal plane (GfSP), and the medial and lateral areas were measured as the volume of the medial (Vmjs) and lateral joint space (Vljs), respectively (Fig. 5d).

### Measurement of the volume of joint space at T1, T2, and T3

Vjs was measured at T1, T2, and T3 after removing the condyles from the glenoid fossa. Because there was no change in Vgf, the value measured at T0 was used. The condyle was removed from the analysis program after confirming the new position by superimposing the postoperatively reconstructed 3D image onto the preoperative 3D image. Vajs, Vpjs, Vmjs, and Vljs were measured using the methods described above.

### Statistical analysis

Data preprocessing was performed using Excel 2016 (Microsoft, Redmond, WA, USA), and SAS version 9.4 (SAS Institute, Cary, NC, USA) was used for analysis and statistical processing.

A linear mixed model and repeated-measures covariance pattern with unstructured covariance were used to evaluate significant changes in the TMJ space volume over time. In the post-hoc analysis, Bonferroni correction was applied, and a *p*-value of 0.0001 or less was considered statistically significant.

The TMJ space volume on day 3 (T1) after surgery was analyzed using a statistically estimated average value using mixed model analysis based on data obtained from 19 patients who underwent CBCT on day 3 (T1) after surgery.

### Ethical approval

The study was approved by the institutional review board of Yonsei University Gangnam Severance Hospital approved this retrospective study (IRB No. 3–2022-0466), and waiver of written informed consent for this retrospective study. This study was also conducted according to the principles of the Declaration of Helsinki for research on humans.

## Data Availability

The datasets used and/or analysed during the current study available from the corresponding author on reasonable request.

## References

[1] Li DTS, Wang R, Wong NSM, Leung YY (2022). Postoperative stability of two common ramus osteotomy procedures for the correction of mandibular prognathism: A randomized controlled trial. J. Cranio-Maxillo-Facial Surg.: Off. Publication Eur. Assoc. Cranio-Maxillo-Facial Surg..

[2] Winstanley RP (1968). Subcondylar osteotomy of the mandible and the intraoral approach. Br. J. Oral. Surg..

[3] Moose S (1964). Surgical correction of mandibular prognathism by intraoral subcondylar osteotomy. J. Oral. Surg..

[4] Hebert JM, Kent JN, Hinds EC (1970). Correction of prognathism by an intraoral vertical subcondylar osteotomy. J. Oral. Surg..

[5] Jung HD, Kim SY, Park HS, Jung YS (2014). Modification of intraoral vertical ramus osteotomy. Br. J. Oral. Maxillofac. Surg..

[6] Manor Y, Blinder D, Taicher S (2001). Intra-oral vertical ramus osteotomy: A modified technique for correction of mandibular prognathism. Int. J. Oral. Maxillofac. Surg..

[7] Bell WH, Yamaguchi Y (1991). Condyle position and mobility before and after intraoral vertical ramus osteotomies and neuromuscular rehabilitation. Int. J. Adult Orthodontics Orthognathic Surg..

[8] Kim JY, You HS, Huh JK, Park KH (2020). Is there a difference in condyle position changing pattern between deviated and non-deviated sides after intraoral vertical ramus osteotomy in facial asymmetry?. J. oral Maxillofacial Surg.: Off. J. Am. Associat. Oral Maxillofacial Surgeons.

[9] Jung S, Choi Y, Park JH, Jung YS, Baik HS (2020). Positional changes in the mandibular proximal segment after intraoral vertical ramus osteotomy: Surgery-first approach versus conventional approach. Korean J. Orthod..

[10] Jung HD, Jung YS, Park HS (2009). The chronologic prevalence of temporomandibular joint disorders associated with bilateral intraoral vertical ramus osteotomy. J. oral Maxillofacial Surg.: Off. J. Am. Associat. Oral Maxillofacial Surgeons.

[11] Ghali GE, Sikes JW (2000). Intraoral vertical ramus osteotomy as the preferred treatment for mandibular prognathism. J. oral Maxillofacial Surg.: Off. J. Am. Associat. Oral Maxillofacial Surgeons.

[12] Kim JY, Kim BJ, Park KH, Huh JK (2016). Comparison of volume and position of the temporomandibular joint structures in patients with mandibular asymmetry. Oral Surg. Oral Med. Oral Pathol. Oral Radiol..

[13] Zhang ZL (2012). Measurement accuracy of temporomandibular joint space in Promax 3-dimensional cone-beam computerized tomography images. Oral Surg. Oral Med. Oral Pathol. Oral Radiol..

[14] Petersson A (2010). What you can and cannot see in TMJ imaging–an overview related to the RDC/TMD diagnostic system. J. Oral Rehabil.

[15] Choi YS, Yun KI, Kim SG (2002). Long-term results of different condylotomy designs for the management of temporomandibular joint disorders. Oral Surg. Oral Med. Oral Pathol. Oral Radiol. Endod..

[16] Ueki K (2007). Condylar and disc positions after intraoral vertical ramus osteotomy with and without a Le Fort I osteotomy. Int. J. Oral Maxillofac. Surg..

[17] Huang L, Tang S, Zou R, Ouyang KX, Piao Z (2021). The three-dimensional evaluation of positional change in mandibular condyle after intraoral vertical ramus osteotomy. J. Stomatol. Oral Maxillofac. Surg..

[18] Nagata Y (2023). Mandibular condylar displacement and the associated factors following intraoral vertical ramus osteotomy. J. Oral Maxillofac. Surg. Med. Pathol..

[19] Hong SUN, Jin-long ZHAO, Li-sheng HE (2020). Three-dimensional reconstruction and comparison of temporomandibular joint space before and after orthognathic surgery. China J. Oral Maxillofac. Surg..

[20] Podčernina J (2020). Evaluation of condylar positional, structural, and volumetric status in Class III orthognathic surgery patients. Medicina.

[21] Ravelo V (2023). Condylar positional changes in skeletal class II and class III malocclusions after Bimaxillary orthognathic surgery. J. Personaliz. Med..

[22] Silva CAGD, Grossi ML, Araldi JC, Corso LL (2023). Can hard and/or soft occlusal splints reduce the bite force transmitted to the teeth and temporomandibular joint discs? A finite element method analysis. CRANIO®.

[23] Ohba S (2015). The three-dimensional assessment of dynamic changes of the proximal segments after intraoral vertical ramus osteotomy. Cranio.

[24] Shi H (2023). Three-dimensional Reconstruction and Comparison of temporomandibular joint space volume before and after orthognathic surgery in patients with skeletal class III malocclusion with mandibular deviation. J. Craniofac. Surg..

[25] Keser E, Naini FB (2022). Accelerated orthodontic tooth movement: Surgical techniques and the regional acceleratory phenomenon. Maxillofac. Plastic Reconstruct. Surg..

[26] Zhao Q, Hu J, Wang D, Zhu S (2007). Changes in the temporomandibular joint after mandibular setback surgery in monkeys: Intraoral vertical versus sagittal split ramus osteotomy. Oral Surg. Oral Med. Oral Pathol. Oral Radiol. Endod..

[27] Dobriyan A (2022). Impact and stability of mandibular setback after intraoral vertical ramus osteotomy. Appl. Sci..

[28] Jung HD, Kim SY, Park HS, Jung YS (2015). Orthognathic surgery and temporomandibular joint symptoms. Maxillofac. Plast. Reconstr. Surg..

[29] Jae-Kwang J, Yun-Kyung H, Jae-Kap C (2011). The Relationship between Temporomandibular joint Pain and the Relative Signal Intensity of Retrodiscal Tissue on T1-, and T2-Weighted MRI Images. JOMP.

[30] Barone S, Muraca D, Averta F, Diodati F, Giudice A (2022). Qualitative and quantitative assessment of condylar displacement after orthognathic surgery: A voxel-based three-dimensional analysis. J. Stomatol. Oral Maxillofac. Surg..

[31] Han S-H, Park JH, Seo HY, Chae J-M (2023). Temporomandibular joint space changes in skeletal class III malocclusion patients with orthognathic surgery. Appl. Sci..

[32] Lim YN, Park I-Y, Kim J-C, Byun S-H, Yang B-E (2020). Comparison of changes in the condylar volume and morphology in skeletal class III deformities undergoing orthognathic surgery using a customized versus conventional miniplate: A retrospective analysis. J. Clin. Med..

[33] Gossi DB, Gallo LM, Bahr E, Palla S (2004). Dynamic intra-articular space variation in clicking TMJs. J. Dent Res..

[34] Quon JL (2020). Artificial intelligence for automatic cerebral ventricle segmentation and volume calculation: A clinical tool for the evaluation of pediatric hydrocephalus. J. Neurosurg. Pediatr..

[35] Pellerin O (2013). Comparison of semi-automatic volumetric VX2 hepatic tumor segmentation from cone beam CT and multi-detector CT with histology in rabbit models. Acad. Radiol..

[36] Trindade JL (2021). Low-dose multidetector computed tomographic and cone-beam computed tomographic protocols for volumetric measurement of simulated periapical lesions. J. Endod..

[37] Tel A (2023). Systematic review of the software used for virtual surgical planning in craniomaxillofacial surgery over the last decade. Int. J. Oral Maxillofac. Surg..

[38] Chung SW, Kim SM, Byun SS, Park HS, Jung YS (2011). Comparative analysis of the reference lines on Mcnamara`s and Delaire`s analyses for the anterior and posterior facial relationship of maxillofacial deformity. Maxillofac. Plastic Reconstruct. Surg..

